# Quinine in Acute Rheumatism

**Published:** 1848-05

**Authors:** 


*Quinine in Acute Rheumatism.*—Most of our readers are probably aware that among other remedies for rheumatic fever, sulphate of quinine has been recommended; a mode of treatment contrasting strongly with that by profuse bleeding, or that by mercury, and yet one, it would appear, very successful. The quinine plan of treatment is followed by M. Trosseau, of the Hôpital Necker. The following is from a clinical lecture:—In a case recently in that hospital, —one of rheumatic fever, with effusion into the knee-joint, but without any cardiac affection,—sulphate of quinine was administered, unpreceded by any other treatment. In four days a marked amelioration took place; from fifteen to thirty grains of the medicine being taken per day. In a few days more the patient was able to walk; all effusion and fever had disappeared. Here, then, was a rapid cure of rheumatic fever: however, that disease mostly lasts for two or three weeks. When it proceeds in its usual manner it may be combated in the same way as intermittent fever. If the quinine be suspended as soon as an evident benefit is produced, there is a danger of relapse, just as when that medicine has arrested an ague fit. The quinine must therefore be given every two, three, or four days, and continued after the pains have ceased. We must enter into the belief that rheumatism is not a local affection, and that an individual is not cured when the pains are stopped. Whilst the patient sweats, whilst his appetite does not return, and there is something wrong in his pulse, the rheumatic diathesis must be treated. When we employ blood-letting, we do not treat the rheumatism; we are only occupied with the diathesis by virtue of which the solids and liquids take on the rheumatic affection. When we make use of quina, we act upon the entire economy, on that particular condition of it known as the diathesis, whence come the pains and other phenomena presented by the joints. The diathesis may persist for two or three years after all manifestation of the disease has ceased, and we are obliged to act against this diathesis as we do in the case of the syphilitic or scrofulous. Such a persistence, or latency, is also seen in the marsh miasm, where a person having once been in an infected locality, may, at an interval of years, get an attack of ague, although in a district quite free from the visitations of that malady. Thus we see that a patient may be cured of all the outward symptoms of a disease, and yet the germs of that disease lurk, as it were, within him, ready to be developed, and to manifest themselves, whenever, from a concurrence of circumstances, they are kindled to a new life. So also, as in ague, the germs of a disease may be imbibed into the system, and lie hidden for a long period; but yet retain their power of being developed into evident disease. Such is also seen in the eruptive fevers, and we may suppose the same thing possible in other diseases.—*Ibid.*

